# Scoping Review on Ethical Considerations in Research on the Work–Family Interaction Process

**DOI:** 10.3390/nursrep15020031

**Published:** 2025-01-23

**Authors:** Miguel Valencia-Contrera, Flérida Rivera-Rojas, Jenifer Villa-Velásquez, Daniella Cancino-Jiménez, Solange Vallejos-Vergara, Naldy Febré

**Affiliations:** 1 Faculty of Nursing, Andrés Bello University, Santiago 7591538, Chile; daniella.cancino@unab.cl (D.C.-J.); naldy.febre@unab.cl (N.F.); 2 Department of Nursing, Faculty of Health Sciences, Catholic University of Maule, Curicó 3460000, Chile; frivera@ucm.cl; 3 School of Nursing, Austral University of Chile, Puerto Montt 5090000, Chile; jenifer.villa@uach.cl; 4 Faculty of Health Sciences, Autonomous University of Chile, Santiago 7500912, Chile; s.vallejos1@uandresbello.edu

**Keywords:** ethics, ethics research, ethical analysis, nursing theory, occupational health, work–life balance, nursing

## Abstract

**Background:** The complex nature of the work–family interaction process means special ethical considerations are required in its study. Symphonology can guide ethical analysis in this area, as it pertains to the study of agreements and the elements necessary to form them. **Objective:** Our objective was to analyze the ethical considerations involved in the development of research on the work–family interaction process via symphonological bioethical theory. **Methods:** A scoping review was conducted by consulting the following databases: Web of Science (WoS), SCOPUS, Cumulative Index to Nursing and Allied Health Literature (CINAHL), PubMed, and Business Source Ultimate. Given the small number of studies identified in the field, we did not discriminate by years of publication and included articles of any design that addressed ethical considerations in research on the work–family interaction process or that were related to the topic, including manuscripts in Spanish, English, and Portuguese. **Results:** The ethical analysis of research on the work–family interaction process requires us to consider the participant’s multi-role status as a “worker,” including their inherent relationships with their environment, such as colleagues and supervisors, and as a member of a “family” unit. The various factors involved in the work–family interaction “context” must be analyzed within the context of situation, knowledge, and awareness. Based on the review findings, a list of recommendations was developed focused on planning, data collection, and result presentation. Key points include the provision of psychological support when the research involves sensitive data; the notification of authorities upon identifying offenses such as workplace abuse or domestic violence; and ensuring confidentiality of participation. **Conclusions:** This review provided answers to the proposed objective, concluding that the symphonological nursing bioethics theory, through its conception and statements, guides researchers to make decisions in the context of research development in the work–family interaction process.

## 1. Introduction

Workers are exposed to a variety of health and safety risks every day, including psychosocial ones [1]. These were highlighted during the COVID-19 pandemic, as there was an increase in research on them [2,3].

Psychosocial risks are defined by a joint committee representing the World Health Organization (WHO) and the International Labor Organization (ILO) as “interactions between work, its environment, job satisfaction and the conditions of its organization, on the one hand, and, on the other hand, the worker’s capabilities, needs, culture and personal situation outside work, all of which, through perceptions and experiences, can influence health and job performance and satisfaction” [4].

Among the types of psychosocial risk, the work–family conflict has been highlighted as the only one that considers the personal family dimension of the worker and has been characterized as one of the least explored [3] and the most filled with theoretical ambiguities in its approach [5]. Recently, a bibliometric study of the 100 most cited studies on conflicts published in the category of nursing in the Web of Science database highlighted “work-family conflict” amongst the most frequently used keywords [6]. Along these lines, it can be affirmed that it has been a phenomenon of interest for the scientific community and will continue to be so at least in the near future, and so it is urgent to understand the ethical considerations in this approach.

Ethics corresponds to a branch of philosophy, and, according to the International Council of Nurses (ICN), it helps to determine the “duty at the social, community or individual level” [7]. In the context of occupational health, there are situations that generate conflict with the obligations of protection towards workers that employers must comply with. These arise from national laws, international treaties, or codes of ethics. When actions or omissions negatively influence the welfare of workers, they become a source of moral reflection on ethics as a pillar in risk prevention [8].

In this sense, the symphonological bioethics theory of Husted and Husted is presented as an opportunity to direct the analysis of this phenomenon [9]. According to the authors, symphonology corresponds to the study of agreements and the elements necessary to form agreements. In its ethical dimension, this focuses on commitments and obligations between involved parties. In research studies, the symphonological approach has proven to be useful, highlighting the explicit nature of the researcher–subject relationship [10].

Along these lines, considering the aforementioned, the present study was conceived with the objective of analyzing the ethical considerations involved in the development of research on the work–family interaction process via symphonological bioethical theory.

## 2. Materials and Methods

A scoping review was performed based on the PRISMA-ScR (PRISMA Extension for Scoping Reviews) standards [11] (Supplementary Material). The question guiding the research was as follows: What are the ethical considerations involved in conducting research on the work–family interaction process?

We consulted the Web of Science (WoS) [including KCI-Korean Journal Database, Russian Science Citation Index and Scielo Citation Index], SCOPUS, Cumulative Index to Nursing and Allied Health Literature (CINAHL), PubMed, and Business Source Ultimate databases.

Regarding the construction of the search equation, the thesauruses Descriptors in Health Sciences (DeCS) and Medical Subject Headings (MeSH) were consulted for the selection of descriptors; pilot tests were carried out with various equations using different descriptors and Booleans. The central equation that proved to be the most effective as well as the search strategies and filters used in each database are shown in Table 1.

### 2.1. Eligibility Criteria

Regarding the study period, due to the small number of studies identified in the field, we did not discriminate by years of publication, and data extraction was performed on 26 May 2024. Regarding the selection criteria, articles that addressed ethical considerations in research on the work–family interaction process or that were related to it, and manuscripts in Spanish, English, and Portuguese, were included, without discriminating by design. Exclusion criteria were letters to the editor, editorials, theses, and conference abstracts.

### 2.2. Information Selection Process

The identified articles were submitted to the Rayyan platform for analysis and the elimination of duplicates (performed by the principal investigator). The studies were then read in abstract form and, subsequently, in a full-text format, applying previously defined inclusion and exclusion criteria (performed by three reviewers, independently); differences in opinions were discussed by the team of researchers, moderated by the principal.

### 2.3. Conducting a Search

**Figure 1:**
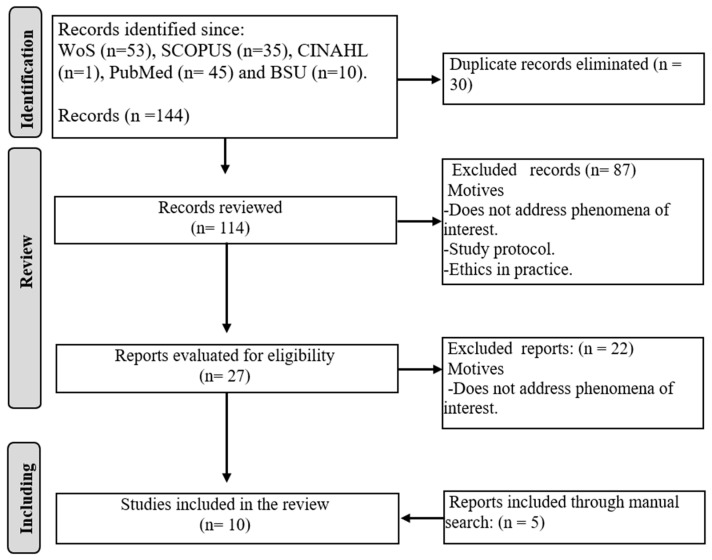
Review flowchart.

After the execution of the search, the articles that successfully passed the filters and the application of inclusion and exclusion criteria corresponded to 5 manuscripts. Due to the limited number of studies, a manual search was conducted using the Google Scholar search engine. Consequently, the final sample consisted of 10 studies [12,13,14,15,16,17,18,19,20,21]. The review flow diagram is presented in Figure 1.

### 2.4. Data Extraction Process

The data were extracted and presented in an Excel document to organize the review process. Descriptive data were extracted from the title of the article, the authors, the year of publication, the language, the journal, the type of article, and the unit of analysis.

### 2.5. Analysis of the Quality of the Studies

Regarding the analysis of the methodological quality of the identified studies, this was not considered due to the nature of this scoping review. Specifically, this was not considered because the focus of interest of the authors corresponds to the ethical aspects related to the investigation of the work–family interaction process, and it is not related to the evidence generated from the studies.

### 2.6. Synthesis Strategy

The data were synthesized using the nursing theory “symphonological bioethical theory”, and we used the model for bioethical decision making as a structure [22].

### 2.7. Ethical Considerations

Regarding ethical considerations, the authors state that the article was developed following the ethical recommendations of good scientific practice, adhering to the principles of honesty, objectivity, integrity, precaution, openness, and responsibility [23]. The authors also declare that they ensure the proper attribution of authorship.

## 3. Results

### 3.1. Characteristics of Included Studies

Regarding the characteristics of the included studies, 80% (n = 8) were published in English, 50% (n = 5) were reflective studies, 50% (n = 5) used the literature on the field as the unit of analysis, and 30% (n = 3) were published in the Journal of Business Ethics. The detailed characteristics of the studies are presented in Table 2.

### 3.2. Description of the Theoretical Elements

In the context of the investigation of the work–family interaction process, the participants not only corresponded to the workers, but also to those in their close environment, such as co-workers and bosses, and the workers’ family members. These individuals are considered as rational beings with a unique character structure in the theoretical framework.

The agreements established between the responsible researcher and the participants are aimed at maintaining and improving the lives of the participants. As for the context, this encompasses the various facts of a situation according to the authors [22]. Three types can be distinguished: the context of the situation, the context of knowledge, and the context of an individual’s consciousness. Therefore, in the present analysis, the context of the situation involves the worker’s work environment and personal family environment; the context of knowledge includes the participant’s pre-existing knowledge of the work–family interaction process; and, finally, the context of awareness constitutes the integration of the participants’ awareness of their situation and their knowledge.

This is consistent with the situationist ethical perspective, which advocates a contextual analysis of actions and the consideration of work–life balance practices and initiatives that benefit all parties [13].

The analysis of the context should not only consider the availability of family policies in the workplace, as this concept has been described as substantially differing from accessibility, i.e., whether employees can actually use the policies where they are available. This highlights the importance of considering these points separately [14]. The “agreements” are developed towards a goal. Decisions on study participation correspond to the result of the dynamic interaction of the elements described.

### 3.3. Bioethical Standards

Bioethical standards constitute a frame of reference in ethical behavior. They include the following attributes: autonomy; doing good and avoiding evil (beneficence); fidelity; freedom; objectivity. Symphonological theory holds that participants are entitled to receive the benefits contained in the bioethical standards, which are described below in Table 3.

Based on the findings of the review, a list of recommendations associated with the development of research on the work–family interaction process has been developed [17,18,19,20,21]. Some correspond to generic elements, and others are specific to the phenomenon of interest (see Table 4).

The proposed list of recommendations can be utilized in the development of research related to the work–family interaction, for instance, in the construction of research protocols. Furthermore, it will prove beneficial for reviewers, editors, and members of scientific ethics committees in the field.

## 4. Discussion

Research focused on the work–family interaction process involves various ethical considerations in its development, since the nature of the phenomenon involves a trilogy of interaction (work, family, and personal dimensions of the worker) that forces researchers to make decisions that transcend the conventional.

Along these lines, symphonological bioethical theory is presented as an alternative in its discussion, which has proven to be of great help in the ethical analysis of various situations, such as in the analysis of humanized care during childbirth [24], in the analysis of the bioethical conflicts that emerge from the care provided by nursing professionals in the management of pain in palliative oncological care patients [25], and others [26].

Symphonology is applicable at all levels of nursing practice [22], and, with no exception made in the case of research on the work–family interaction process, is able to guide decisions that are centered on the participants and ethically justified. The theory invites researchers to consider the respect and recognition of the participants, to see them as unique beings who have the right to receive the benefits contained in bioethical norms (autonomy, fidelity, freedom, objectivity, doing good and avoiding evil), and to consider the context of a concrete situation, which highlights the individualized character of each decision.

The study of the family, per se, constitutes a complex research unit; the same applies to the study of work life. Therefore, when an interaction between both elements is added, the complexity becomes synergistic. Regarding work, due to its diverse nature and the conditions and regulations of each country, researchers face different ethical challenges in the development of studies in the field. Along this line, Iavicoli et al. [27] emphasize their own experience and knowledge of and competencies in professional training in occupational health, as well as in multidisciplinary collaborative work, as strong points in contextualizing ethical choices.

On the one hand, immersing oneself in the personal family privacy of each worker is a long-standing problem, and reviews of the literature in the field show the need for future research focused more on the family aspect of work–family conflict [28,29]. In response to this, proposals have arisen to help reconcile ethical tensions, for example, Dubois et al. [30] emphasize reflexivity, flexibility, adaptability, and participation by questioning children on their family life.

However, despite the complexity of the phenomenon, it is necessary to promote its study. Otherwise, the copious and varied harmful effects of conflict between the work and family sphere of workers will be perpetuated, affecting attitudes and behaviors at work (e.g., absenteeism), family dynamics (e.g., parental conflicts), personal and family health (e.g., physical and mental health problems), and factors associated with work products (e.g., customer satisfaction) [28,31,32].

Finally, based on the findings of this study, the scientific community is invited to recognize the complexity of the phenomenon, which requires those interested in the field to confront specific challenges. While the symphonological bioethical theory underscores the unique nature of each decision, we propose that the analysis should be centered on a triad: work, family, and personal dimensions of the worker.

### 4.1. Limitations

The present study is not free of limitations. As a secondary analysis, the results are consistent with the search strategies. This could be improved, for example, by including gray literature. However, this is the first proposal in the field, of which we are aware, to invite researchers to ethically consider the investigation of the work–family interaction process. Along these lines, as a consideration for future studies, we recommend the development of the discussion around the associated ethical dilemmas. Also, it would be interesting to know the experiences of the researchers and of the scientific ethical committees that have been in front of the phenomenon of interest. The work of these heterogeneous artists will allow us to generate more solid recommendations in the field.

### 4.2. Implications for Nursing Research

Nursing has great challenges in addressing the phenomenon of interest. For instance, on the one hand, in the health sector, this is an occupational group particularly affected by the work–family conflict [33]. Therefore, it is expected that the scientific community will continue to develop research in the field; however, despite being a relevant phenomenon that it is necessary to understand, due to its nature, research into it is not an easy task in practice, and for this reason the present study highlights the need to give special consideration to planning, data collection, and even the presentation of results.

## 5. Conclusions

The present review provided answers to the proposed objective, concluding that the symphonological bioethical theory of nursing, through its conception and statements, guides researchers to make decisions in the context of research development in the work–family interaction process.

Using the model for bioethical decision making, the context in which the studies associated with the phenomenon are developed is taken into account, and this has implications in a transversal manner. Along these lines, recommendations are provided for the development of studies in the field, considering planning, data collection, and presentation of results.

Future studies could contribute to the field by sharing the experiences of more experienced researchers, as each investigation encounters unique challenges whose approaches will undoubtedly provide valuable insights to the scientific community.

## Figures and Tables

**Table 1:** Central search equation, strategy, and filters applied.

Central Search Equation	
(((“work-home Interference”) OR (“work-family interface”) OR (“work/family Balance”) OR (“work-family interaction”) OR (“work-family conflict”) OR (“work-family tension”) OR (“work-life conflict”) OR (“work-family spillover”) OR (“work-family spillover”) OR (“home-work Interference”) OR (“family-work interface”) OR (“family/work Balance”) OR (“family-work interaction”) OR (“family-work conflict”) OR (“family-work tension”) OR (“life-work conflict”) OR (“family-work spillover”)) AND ((Ethics) OR (“Ethical Issues”) OR (“Ethical Issue”) OR (“Issue, Ethical”) OR (“Issues, Ethical”) OR (“Situational Ethics”) OR (“Ethics, Situational”) OR (“Ethics, Research”) OR (“Research Ethics”) OR (“Eth-ical Review”) OR (“Review, Ethical”) OR (“Ethics Committees”) OR (“Ethics Committees, Research”) OR (“Committee, Research Ethics”) OR (“Committees, Research Ethics”) OR (“Boards, Institutional Review”) OR (“Institutional Re-view Boards”) OR (“Review Boards, Institutional”) OR (“Review Board, Institutional”) OR (IRB) OR (“Research Ethics Committees”) OR (IRBs) OR (“Research Ethics Committee”) OR (“Ethics Committee, Research”) OR (“Board, Institution-al Review”) OR (“Institutional Review Board”) OR (“Ethical Review”) OR (“Re-view, Ethical”) OR (“Ethics, Professional”) OR (“Ethics, Institutional”) OR (“Ethical Analysis”)))	
Database	Search Strategy
WoS	All fields
SCOPUS	Article title, abstract, keywords
CINAHL	AB Summary
PubMed	All fields
Business Source Ultimate	AB Summary

**Table 2:** Characteristics of included studies.

Title, Authors and Year of Publication	Language	Journal	Type of Article	Unit of Analysis
Conciliating Work and Family: A Catholic Social Teaching Perspective (Guitián G, 2009) [12].	English	*Journal of Business Ethics*	Reflection	Literature in the field
A Discovery of Early Labor Organizations and the Women who Advocated Work-Life Balance: An Ethical Perspective (Phipps STA and Prieto LC, 2016) [13].	English	*Journal of Business Ethics*	Reflection	Literature in the field
Family-supportive workplace policies and South Korean mothers’ perceived work-family conflict: accessibility matters (Kim EJ and Parish SL., 2020) [14].	English	*Asian Population Studies*	Quantitative approach	Data from the Korean Longitudinal Survey of Women and Families
Work-Family Conflict: A Virtue Ethics Analysis (Marchese M., et al., 2002) [15].	English	*Journal of Business Ethics*	Reflection	Literature in the field
Morality and Work-Family Conflict in the Lives of Poor and Low-Income Women (Hennessy J., 2009) [16].	English	*The Sociological Quarterly*	Qualitative approach	Semi-structured interviews with poor and low-income women.
Etica de la investigación psicosocial (Mondragón Barrios L., 2007) [17].	Spanish	*Salud mental*	Reflection	Literature in the field
Consideraciones éticas en la investigación etnográfica institucional (Valencia-Contrera, M., 2023) [18].	Spanish	*Persona y bioética*	Integrative review	Master’s and Doctoral Theses
Ethical considerations when conducting joint interviews with close relatives or family: an integrative review (Voltelen, B. et al., 2018) [19].	English	*Scandinavian Journal of Caring Sciences*	Integrative review	Scientific articles
Research with bereaved families (Sque M., et al., 2014) [20].	English	*Nursing Ethics*	Qualitative approach	Semi-structured interviews with participants from 31 families
Protecting the Privacy of Family Members in Survey and Pedigree Research (Botkin, J.R., 2001) [21].	English	*JAMA*	Reflection	Literature in the field

**Table 3:** Bioethical standards of symphonological bioethical theory.

Standard	Description	Association
Autonomy	The uniqueness of the individual, the singular character structure of the individual. Every person has the right to act according to his unique and independent purposes [10].	Organizations should promote autonomy in the process of coordinating and integrating work and personal aspects of lives; therefore, they should make decisions in this regard [13].
Doing good and avoiding evil (beneficence)	Beneficence is the ability to act to achieve desired benefits and vital needs. Each person can act to obtain what they need and prefer [10].	When immersed in the study of the reality of a job and when conflict is identified between the work and family sphere of workers, it is imperative to identify the sources of such conflict for the implementation of solutions, meaning that researchers must consider what the results will be used for. The identification of problems raises ethical responsibilities [12]. However, the implementation of strategies can only be decided contextually, as Marchese, et al. [15] point out. As such, in the case where the implementation of favorable policies to achieve work–family reconciliation is beneficial for employees and employers, the ethical call is easy, since the policies must be adopted. Likewise, if the implementation of policies threatens the long-term financial health of the company, it is also an easy ethical call, as the policies should not be adopted, since no one will benefit if the company goes bankrupt. However, in the event that the policies reduce the company’s profits, but not to the point of seriously threatening economic stability, ethical conflicts arise. Considering virtue ethics, their end results should include human rights.
Fidelity	An individual’s loyalty to his or her own uniqueness. All individuals have the right to maintain, manage, and improve their lives. For the health care professional, loyalty to the agreement implies commitment to the obligations accepted in the professional role [10].	The researcher must ensure compliance with what is stated in the research project, as well as comply with what is stated in the informed consent. On the other hand, it should be considered as a norm with shared responsibilities, where the scientific ethical committees should control the compliance of the researchers.
Freedom	This entails the ability and right to act based on the agent’s own assessment of the situation. Each person may choose their course of action without interference [10].	Hennessy [16] states that choices in the work and family sphere are morally charged and linked to cultural structures, which frame self-understanding and philosophies of life.
Objectivity	This entails the right to have an objective conscience and to act accordingly. Each person has a consciousness and a perception of the universe that exists outside of themselves. Each person has the right to manage, maintain, and sustain this understanding as they choose [10].	The responsible investigator should respect the participant’s decision regarding interest in the study, explicitly stating that they may voluntarily withdraw from the study and that no explanations should be given if not considered necessary.

**Table 4:** Recommendations for the development of research on the work–family interaction process.

**Planning**
The research must be approved by the participating institutions. The research must be approved by a scientific ethics committee. The selection of participants should be confidential, either following an individual strategy (each collection separately), or as a whole (analysis by worker-family unit). The delivery of study information according to the participant (employee, management, or family member).
**Data Collection**
5. The signing of informed consent should be considered as a process. 6. In the case of considering the participation of minors, there must be the consent of the legal guardian and assent from the minor. 7. The date, time, and place of data collection should be coordinated with participants (e.g., off-site). 8. Offer participants the option of referral for psychological support, should they feel distressed when discussing and exploring their personal experiences in the institution. 9. Notification in case of inquiry of highly sensitive content (for example, labor abuse, domestic violence, among others). 10. Ensure the security of the data collected (e.g., storage of documents in the personal office of the responsible researcher or digital storage of documents with a password).
**Presentation of Results**
11. In the event that the findings are negative, communicate them to the organization before making them public, following the process defined in the research protocols. 12. On the other hand, the dissemination of the results must ensure that they reach beyond the academic community, including families, along with professionals. 13. Retribution of information to the researched community. 14. Avoid exposing the vulnerability of participants.

## Data Availability

The raw data supporting the conclusions in this article will be made available by the authors without undue reservation, to any qualified researcher.

## Supplementary Materials

The following supporting information can be downloaded at: https://www.mdpi.com/article/10.3390/nursrep15020031/s1, PRISMA-ScR (PRISMA Extension for Scoping Reviews) (Supplemental Material).

## Public Involvement Statement

No public involvement in any aspect of this research.

## Guidelines and Standards Statement

This manuscript was drafted against the PRISMA Extension for Scoping Reviews [11].
